# Learning the Treatment Process in Radiotherapy Using an Artificial Intelligence–Assisted Chatbot: Development Study

**DOI:** 10.2196/39443

**Published:** 2022-12-02

**Authors:** Nathanael Rebelo, Leslie Sanders, Kay Li, James C L Chow

**Affiliations:** 1 Department of Physics Toronto Metropolitan University Toronto, ON Canada; 2 Department of Humanities York University Toronto, ON Canada; 3 Department of English University of Toronto Toronto, ON Canada; 4 Radiation Medicine Program Princess Margaret Cancer Centre University Health Network Toronto, ON Canada; 5 Department of Radiation Oncology University of Toronto Toronto, ON Canada

**Keywords:** chatbot, artificial intelligence, machine learning, radiotherapy chain, radiation treatment process, communication, diagnosis, cancer therapy, internet of things, radiation oncology, medical physics, health care

## Abstract

**Background:**

In knowledge transfer for educational purposes, most cancer hospital or center websites have existing information on cancer health. However, such information is usually a list of topics that are neither interactive nor customized to offer any personal touches to people facing dire health crisis and to attempt to understand the concerns of the users. Patients with cancer, their families, and the general public accessing the information are often in challenging, stressful situations, wanting to access accurate information as efficiently as possible. In addition, there is seldom any comprehensive information specifically on radiotherapy, despite the large number of older patients with cancer, to go through the treatment process. Therefore, having someone with professional knowledge who can listen to them and provide the medical information with good will and encouragement would help patients and families struggling with critical illness, particularly during the lingering pandemic.

**Objective:**

This study created a novel virtual assistant, a chatbot that can explain the radiation treatment process to stakeholders comprehensively and accurately, in the absence of any similar software. This chatbot was created using the IBM Watson Assistant with artificial intelligence and machine learning features. The chatbot or bot was incorporated into a resource that can be easily accessed by the general public.

**Methods:**

The radiation treatment process in a cancer hospital or center was described by the radiotherapy process: patient diagnosis, consultation, and prescription; patient positioning, immobilization, and simulation; 3D-imaging for treatment planning; target and organ contouring; radiation treatment planning; patient setup and plan verification; and treatment delivery. The bot was created using IBM Watson (IBM Corp) assistant. The natural language processing feature in the Watson platform allowed the bot to flow through a given conversation structure and recognize how the user responds based on recognition of similar given examples, referred to as intents during development. Therefore, the bot can be trained using the responses received, by recognizing similar responses from the user and analyzing using Watson natural language processing.

**Results:**

The bot is hosted on a website by the Watson application programming interface. It is capable of guiding the user through the conversation structure and can respond to simple questions and provide resources for requests for information that was not directly programmed into the bot. The bot was tested by potential users, and the overall averages of the identified metrics are excellent. The bot can also acquire users’ feedback for further improvements in the routine update.

**Conclusions:**

An artificial intelligence–assisted chatbot was created for knowledge transfer regarding radiation treatment process to the patients with cancer, their families, and the general public. The bot that is supported by machine learning was tested, and it was found that the bot can provide information about radiotherapy effectively.

## Introduction

### Rationale

To educate patients with cancer, their families, and the general public about the radiation treatment process in a cancer hospital or center, understanding the radiotherapy chain, including all the steps that a patient visiting the clinic has to go through, is necessary. For those people who are unfamiliar with radiotherapy, the radiotherapy chain is complex and intimidating [1,2]. By developing a simple and comprehensive internet-based virtual assistant such as chatbot or bot through which the radiation treatment process can be explained to the user at their own pace, the user can be guided through each step in the radiotherapy chain with simple and relevant information. This was the basis for creating a chatbot capable of explaining this process and reducing the risk of misinformation for the user, while also benefiting from availability [3,4]. Patients must schedule an appointment with the radiation oncologist, who has a fixed period during which they can ask questions, but a bot will be permanently available to them.

Although the bot is functional for purposes of explaining radiotherapy chain to the users, it serves as a proof of concept for more sophisticated and open-ended health care chatbot. The IBM Watson application programming interface (API) allows the programmer to easily add to the program and update any connected applications automatically [5]. Alternatively, additional dialogue chains can be added into a single assistant, which could be used to combine a general assistant such as this one with more specialized assistants. For example, one could focus on a specific part of the radiotherapy chain and can respond to queries specific to a single portion.

### Background

#### The Radiotherapy Chain

The radiotherapy chain refers to the various steps during a patient’s treatment, from the initial diagnosis until their eventual radiation treatment in the cancer hospital or center. Generally, the steps in the radiotherapy chain consist of the major checkpoints in the radiation treatment. In this bot, the radiotherapy chain includes seven key stages in the following order: (1) patient diagnosis, consultation, and prescription; (2) patient positioning, immobilization, and simulation; (3) 3D imaging for radiation treatment planning; (4) target volume and organ delineation; (5) radiation treatment planning; (6) patient setup and plan verification; and (7) treatment delivery. First, the bot will provide a big picture of the radiotherapy chain to the user and state these 7 steps before explaining each of them in detail.

The first step in the chain is diagnosis, consultation, and provision of prescription. This is when the radiation oncologist meets the patient with cancer and conducts a medical examination. After reviewing the patient’s medical record, laboratory test result, and radiology report, the radiation oncologist decides whether radiotherapy is suitable for the patient. If this is the case, the radiation oncologist will prescribe radiation dose for the tumor and prepare the patient for the next step of patient positioning, immobilization, and simulation in the chain. This step, along with the next step, 3D imaging for radiation treatment planning, are sometimes combined and referred to as a single *patient simulation* step. The purpose of these steps is to acquire the patient’s 3D image set of anatomy including the tumor through computed tomography (CT) for treatment planning. This involves positioning the patient on the treatment couch such that they can be seated comfortably, while at the same time, the radiotherapists are able to place the patient in the same position in daily treatment. As even a minor positional or orientation deviation would change the radiation beam targeting during irradiation, a variety of immobilization devices such as headrests and body molds are used to ensure minimal movement [6]. After the patient has been immobilized, a CT scan is performed to acquire the information about the patient’s anatomy using a CT simulator. This allows the radiation staff to identify the tumor and critical organs. In some cases, a contrast agent will be injected into the patient to improve the quality of the scan [7]. Once the tumor has been identified and its size and shape have been determined, the patient is usually marked on the treatment couch, to indicate the targeted radiation dose delivery in the final step of the radiotherapy chain. For the acquired CT image set of the patient, the radiation oncologist contours the 3D shape and location of the tumor. This process is becoming more automated with the increasing accuracy of software image recognition and deep learning [8,9]. The tumor is referred to as the gross tumor volume. Once this has been obtained, the clinical target volume and planning target volume are identified [10]. The planning target volume includes portions of healthy tissue, which is simply an inevitable consequence of current radiation treatment. The target volume is the focus of the next step—treatment planning. The main goal is to determine a treatment plan, which delivers a high dose to the target volume, while at the same time, minimizes radiation exposure to the surrounding healthy tissues. Radiation oncologist, radiotherapist, and medical physicist work together to create a plan that is tailored to the treatment based on plan variables such as positions and sizes of the patient, target, and critical organs. After the treatment plan is created, patient setup verification is initiated. This is referred to as verification simulation. In this step, the patient’s treatment is conducted as a *dry run* before the patient’s actual treatment. This process is performed with the help of computers to simulate the patient’s anatomy and cancer location, for both practice and quality assurance purposes [11]. A study conducted in 2018 confirmed that this process resulted in reduction in the rate of incidents during treatment and anxiety levels of both patients and staff during treatment [12]. After patient setup and plan verification, the patient is finally ready to receive radiation treatment. In most cases, the course of treatment includes one to thirty fractions. In the patient’s first fraction, the patient is placed in the position established during their initial immobilization. Radiotherapists deliver the dosage as prescribed by the radiation oncologist. Patient images from cone-beam CT or portal imager are taken at this point to guide the patient setup. This informs the radiation staff about whether the treatment dosage is effective [13,14].

#### Health Care Chatbots

The radiotherapy chain is a lot of information for a patient or family member to process in a single session. It is important for them to understand the basic treatment process when visiting the cancer center, with a similar level of detail as a patient received during briefing from a radiation staff in an in-person education section. One of the most prominent advantages that health care chatbots have over physicians is their accessibility. A chatbot can be accessed by patients at any time through internet. An autonomous health care chatbot can learn from all available information on any given topic in minutes, as opposed to years. All physicians and medical specialists are limited by information gathered through their own experiences or readings. A software bot such as a patient-facing bot can feasibly learn from the experiences of every health care bot and process information on a given topic at a rate incomparable with that of humans [15]. A sufficiently advanced software bot could make connections that no human would be capable of simply owing to the incomparable processing capabilities of modern computers. Health care chatbots are an inevitable consequence of these advancements. They provide a means by which a practically infinite information source can be simplified and communicated to suit the needs of a human [16]. This can be achieved through scripting and computer programming on the chatbot virtual assistant platform. Chatbots for the purposes of health care have existed since the 1960s, but the computers of the time simply lacked both the information and optimization to have any practical use [17]. This was much before computers were capable of natural language processing (NLP) or machine learning (ML). In recent years, many health care chatbots have been adopted for a variety of purposes, ranging from chatbots encouraging physical fitness to diagnostic chatbots [18]. Although health care chatbots have not yet been fully adopted by the medical field, receptiveness toward the idea of increased use of health care chatbots is moderately high and shows signs of continuing to increase simultaneously with improvements in NLP and ML [19,20]. This is especially true for informational health care chatbots, which show high levels of receptiveness among medical professionals. A study in the United Kingdom found high demand for increased chatbot use in health care, especially among young populations [21].

#### Turing Test

**Figure 1:**
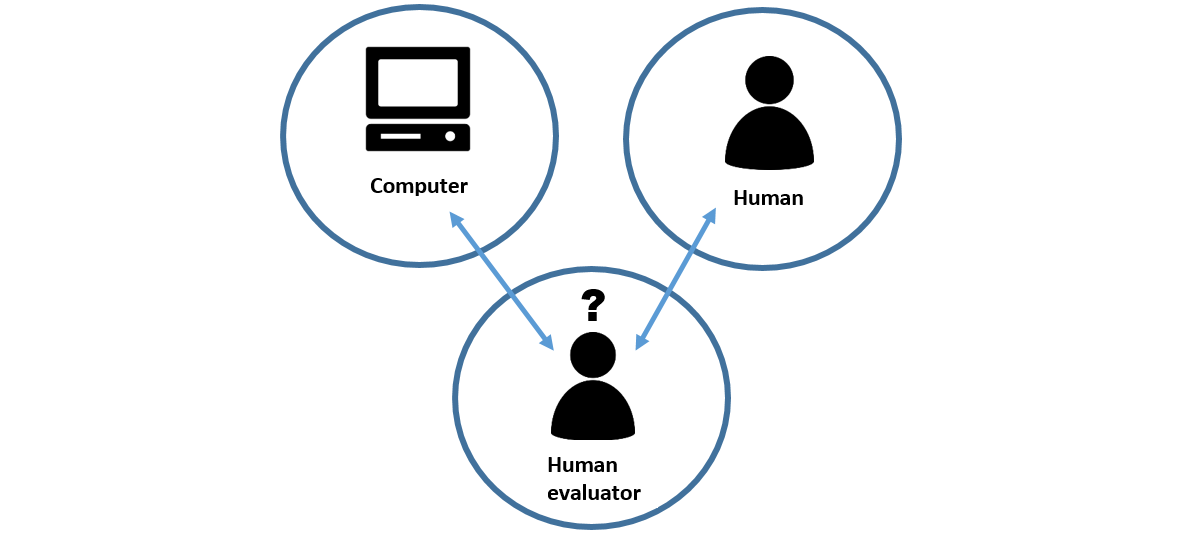
Schematic diagram showing the Turing test. In the test with the human evaluator, in which the human and computer are separate from one another, the human evaluator asks a series of questions to both the human and computer. Then, the evaluator tries to identify which side is operated by the human and which side is operated by the computer.

Turing test was suggested by Alan Turing in 1950 to justify a machine’s ability to exhibit intelligent behavior equal to that of a human [22,23]. In the test, an evaluator would judge the conversations between a human and a machine (computer) designed to mimic human-like responses (Figure 1). The human evaluator, human, and computer are isolated from one another, and the conversation would be limited to the text-only channel. To pass the test, the evaluator must not distinguish the computer from the human. Since the Turing test was introduced, it has become an important concept in the philosophy of artificial intelligence to justify whether the intelligence of a computer is similar to that of a human. The Turing test affects the development of digital communication program such as NLP [24]. ELIZA is an NLP computer program created at the Artificial Intelligence laboratory in Massachusetts Institute of Technology between 1964 and 1966 [25]. ELIZA simulated the conversion with users using the pattern matching approach, which demonstrated superficiality of communication between humans and machines. As people attributed human-like feelings to ELIZA, it is believed that the program could positively influence the lives of many people. The example of ELIZA inspired the idea to create chatbot with artificial intelligence to help users who need human-like touch and communication [15,16].

### Objectives

The goal of this study was to develop a bot capable of explaining the radiation treatment process in terms of radiotherapy chains to the user in a simple and informational manner. Maintaining the bot as less conversational would also benefit from having a reduced risk of uncanny valley effects. Using IBM Watson’s API, a basic conversation structure was created to explain the purpose of a radiotherapy chain and teach the user about each step in the chain. During bot development, it was important to program sufficient number of expected intents for the program to understand what the user is trying to communicate and generate a response. Another goal was to enable the bot to respond to requests for additional information with more detail and, if this was insufficient, direct the user to a source for more in-depth information. Although this bot was designed with various restrictions in mind, an important intention was to allow the bot to be directly implemented into a variety of applications. Many rudimentary chatbots are restricted to a single conversation flow, which makes it time consuming for a user to make requests for information. This bot was created with user efficiency as a priority.

## Methods

### IBM Watson Assistant

#### ML and NLP

The bot was created using IBM Watson Assistant with ML feature. The current state of NLPs allows for Watson to flow through a given conversation structure and recognize how the user responds based on recognition of similar given examples, which are referred to as *intents* during development [26]. Although Watson cannot answer any question posed by the user, it can recognize a given information based on expected input. This is done by receiving a variety of example responses and recognizing similar users’ responses using Watson’s NLP. Once completed, Watson Assistant can be integrated into a variety of different applications or websites depending on its purpose. One of the main advantages of Watson is its API [27], which is simple and user-friendly, allowing for a variety of very simple implementations into commonly used apps such as SMS text messaging and WhatsApp.

#### Watson API

##### Intent

Watson recognizes user input as *intents*. These are values chosen by the programmer based on expected user input. The values are separate from the dialogue chain and are effectively used by Watson as variables. If the chatbot is expected to request information from the user, the programmer provides as many predictable responses as possible to represent a specific user request. Some of these intents will be referred to frequently, whereas others will be referred to only once.

##### Nodes

Basic conversation structure is created using user-created nodes. Every unique node represents a specific section of the dialogue. In Watson, these nodes can be connected either in parallel or sequentially according to the dialogue tree. For example, the dialogue has the potential to split based on user input; then, the node can be created in parallel to another node. If the dialogue is only designed to flow from one point to another, the dialogue will be written in series. Nodes that lead to a series are referred to as parent nodes, and the nodes that follow are referred to as child nodes. Nodes that have no child nodes run in parallel with the first node and are referred to as root nodes. Each dialogue node can be activated by a specific condition—almost always in the form of intents. Once a node is completed, the chatbot has 2 options. It can either wait for a reply if user input is required for the next child node or jump to another node at any point in the dialogue.

##### Digressions

Dialogue nodes have many different options depending on their function in relation to the chatbot. Digression settings allow the programmer to allow or disallow nodes from being activated, when the node exists outside the current dialogue chain. The programmer can use *digression* to set responses that can be activated at any point in time. This is especially effective for developing methods to allow the user to skip certain portions.

##### Multiple Conditioned Responses

Dialogue nodes can also be set to respond to multiple conditions and have varying responses depending on the user input. This is useful for forming responses to conversation-specific questions. A dialogue node can contain responses to multiple questions and then immediately return to the dialogue chain. Without this functionality, questions would need to be represented by individual nodes. Then, the nodes would require *jump to* commands to return to the original dialogue chain or duplicate sets of child nodes to continue the conversation.

##### Testing

The testing feature in the API allows the programmer to examine the program without requiring an integration of the bot. The tester will display the user’s intent when the user makes a statement. This can be used to solve disambiguation issues when the program has >1 similar intent.

### The Radiotherapy Chain Dialogue Tree

**Figure 2:**
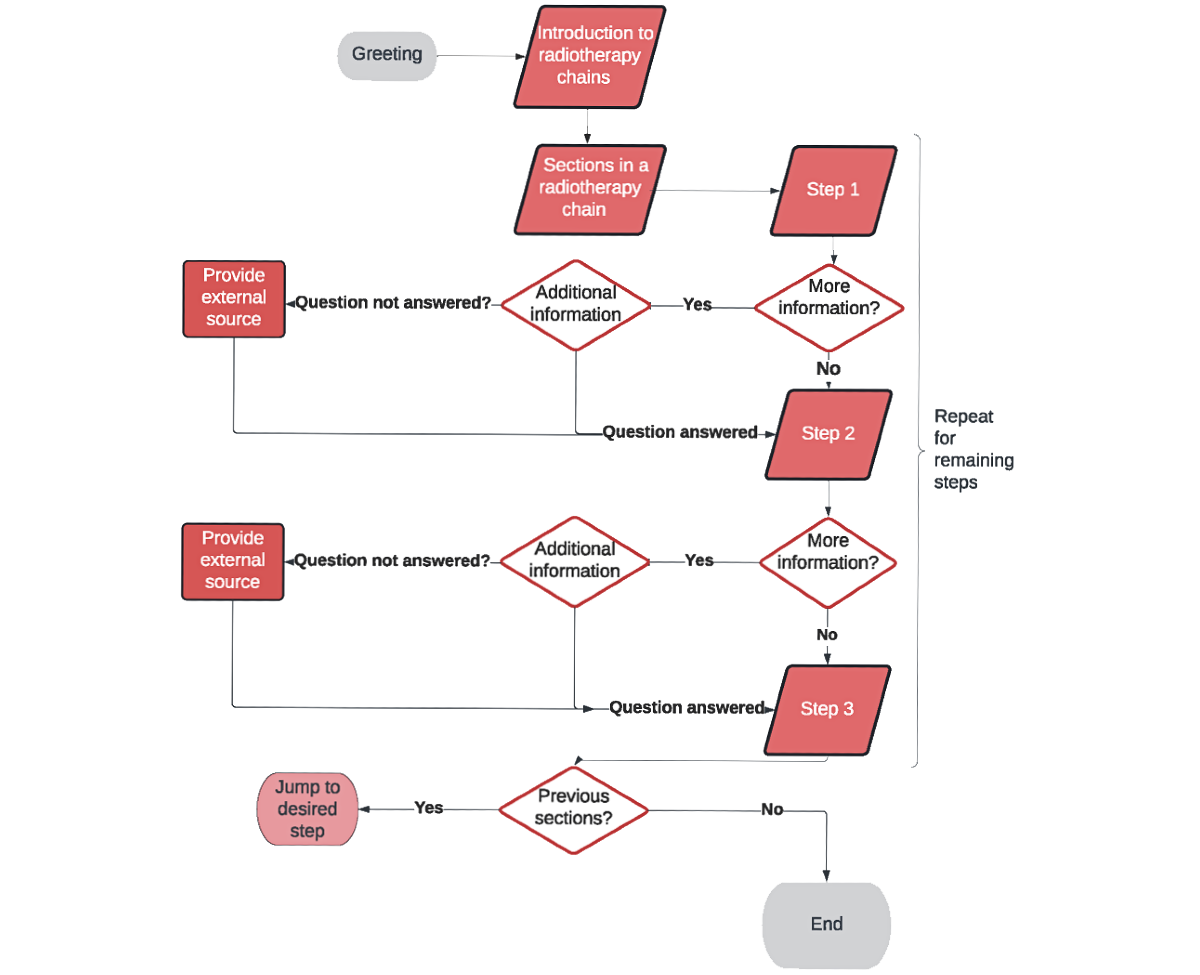
Flowchart of the dialogue chain.

The bot was designed by establishing a dialogue tree based on a series of predefined rules to guide the users by offering them a conditional *if or then* at each step. This conversation flow is based on the workflow of the bot, as shown in Figure 2. The tree was created by the programmer by considering the feedback from the users, and the complexity of the tree depends on the amount of content in the bot. The intent was to create an informational tool capable of being accessed and used by the user in the most efficient manner possible. From this, the basic dialogue tree was formed. This was represented in the Watson platform by a single parent node indicating the start of the conversation, with each step of the radiotherapy chain represented as a child node of the previous step. Once this was finalized, a variety of global variables were created in parallel to the initial parent chain. These were programmed using the *jump to* commands, because they are not part of the conversation structure. They can be accessed at any time if the user asks the bot about a specific section of the radiotherapy chain or if the user simply wants to start from the beginning. At this point, the bot was already functional, but it did not yet satisfy the goal of responding to queries for information. Child nodes were added to each link in the dialogue chain to respond to requests for more information and another child node was added to indicate whether the information was unsatisfactory to the user. Figure 2 shows a flowchart of the dialogue chain. As indicated, the user can either go through the radiotherapy chain by selecting *continue* at each dialogue node. In Figure 2, the nodes in the form of a red rhombus represent nodes along the parent chain, which require user input to continue. The white diamonds represent potential forks in the chain. After each of these forks, the bot will return to the parent chain.

### Integration

For integration, a website was chosen to host the bot for the sake of simplicity. As the topic is relatively specific, integrating it into a website allows the bot to be found by web crawlers. This ensured that if someone looks for a radiotherapy chatbot on a search engine, this bot can easily be found. The website was designed and hosted through Weebly (Block Inc)—a simple website builder that allows for easy-to-use customization options and hosting. The website was designed with simplicity in mind and to serve purely as a site for the bot.

### Bot Testing

The bot was reviewed by the radiation staff and researchers regarding the idea, scope, content, and infrastructure in the cancer center, to fine-tune the bot for the nonexpert users among the general public, including patients and their family members. Specifically, a sample population of 50 people from the general public was asked to use and evaluate the bot and score their experience on a scale ranging from 1 to 5. This was measured based on 3 different metrics: information quality, user experience, and navigability. Upon completion, they were asked to copy and paste their chat logs for review. This was conducted to establish trends in use. These trends could be used to guide further development of the bot.

### Ethics Approval

This study did not require ethics approval because it is only related to the creation of a software of an educational chatbot for information transfer with a performance evaluation and quality assurance. The data in this study are anonymous and deidentified.

## Results

### Overview of the Bot

**Figure 3:**
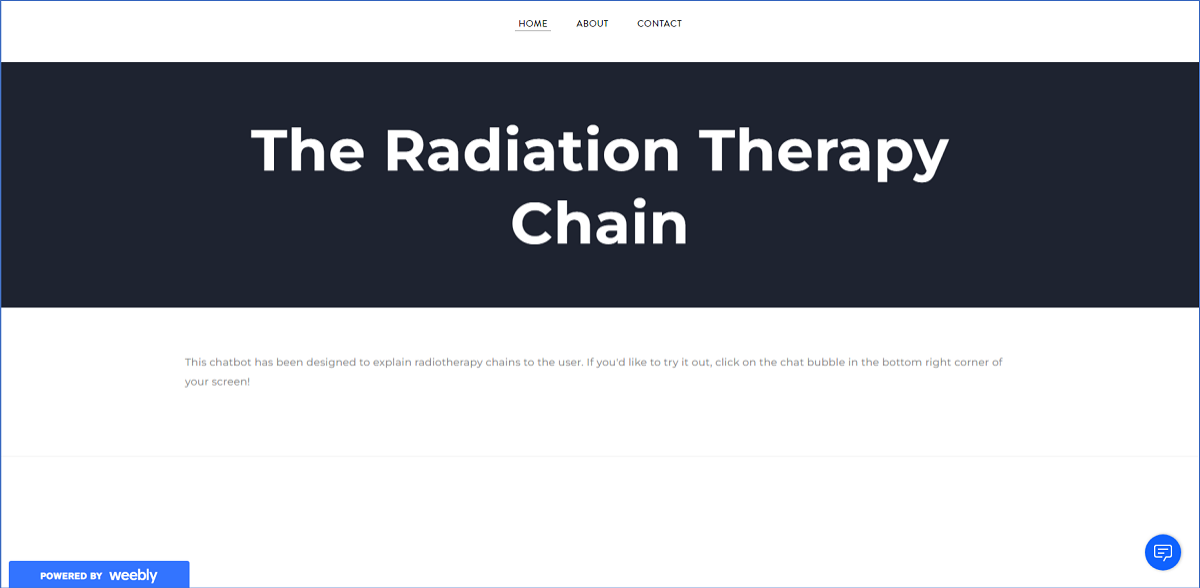
Front-end window of the bot website home page.

The bot can be accessed via the web [28]. Currently, the bot is fully functional. As anticipated, the bot is fully capable of guiding the user through the conversation structure and can respond to simple questions and provide resources for requests of information that were not directly programmed into the bot. The front-end window of the bot is shown in Figure 3.

### Communication Between the Bot and User

**Figure 4:**
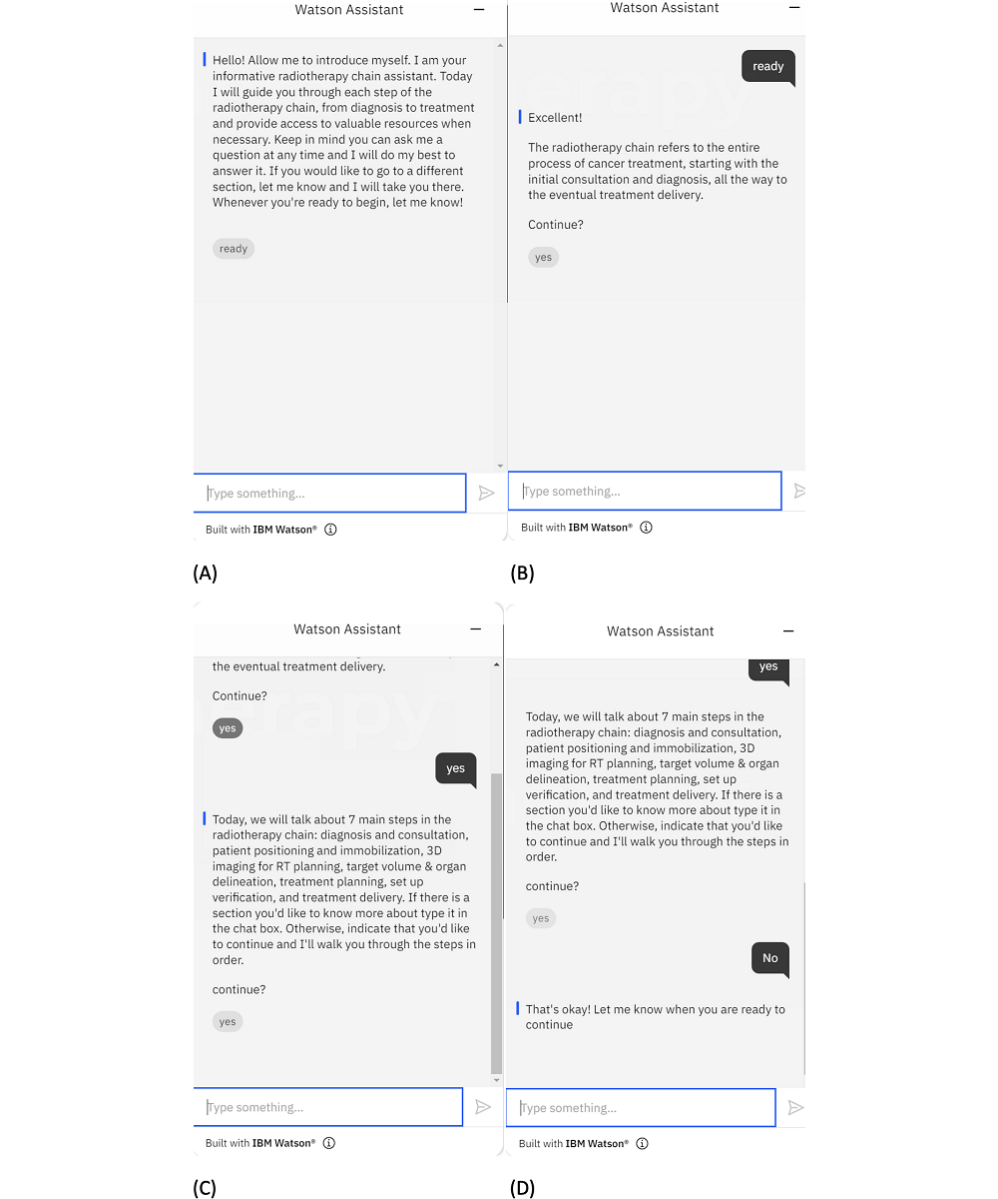
Communications between the bot and the user regarding (A) an introductory message about the bot requiring the user to respond whether they are ready to start, (B) a brief explanation about the radiotherapy chain, and (C) more detailed description of the radiotherapy chain with each step in the process. The user can say “yes” to continue or “no” to discontinue, and (D) when the user answers “no,” the bot will stop until the user is ready to continue.

In Figure 3, the user can start the bot by clicking the chat bubble in the bottom right-hand corner. An introductory message to say hello to the user and explain the functionalities of the bot is shown in Figure 4A. The introductory message informs the user that the bot will guide them to understand the radiotherapy chain step by step. In addition, the user is welcome to ask question at any time and jump to different steps in the chain. When the user is ready, they can type “ready” in the textbox or click the “ready” icon to continue. In this case, the bot will send the next message to explain what a radiotherapy chain in the radiation treatment process is (Figure 4B). The user can answer “yes” to continue, and the bot will inform the user generally about the procedures the patient with cancer will need to follow one by one when they visit a cancer hospital or center (Figure 4C). When the user understands a step, they can continue to the next step. However, the user can answer “no” to the bot. In this case, the bot will request the user to inform when they will be ready to continue. This is shown in Figure 4D.

**Figure 5:**
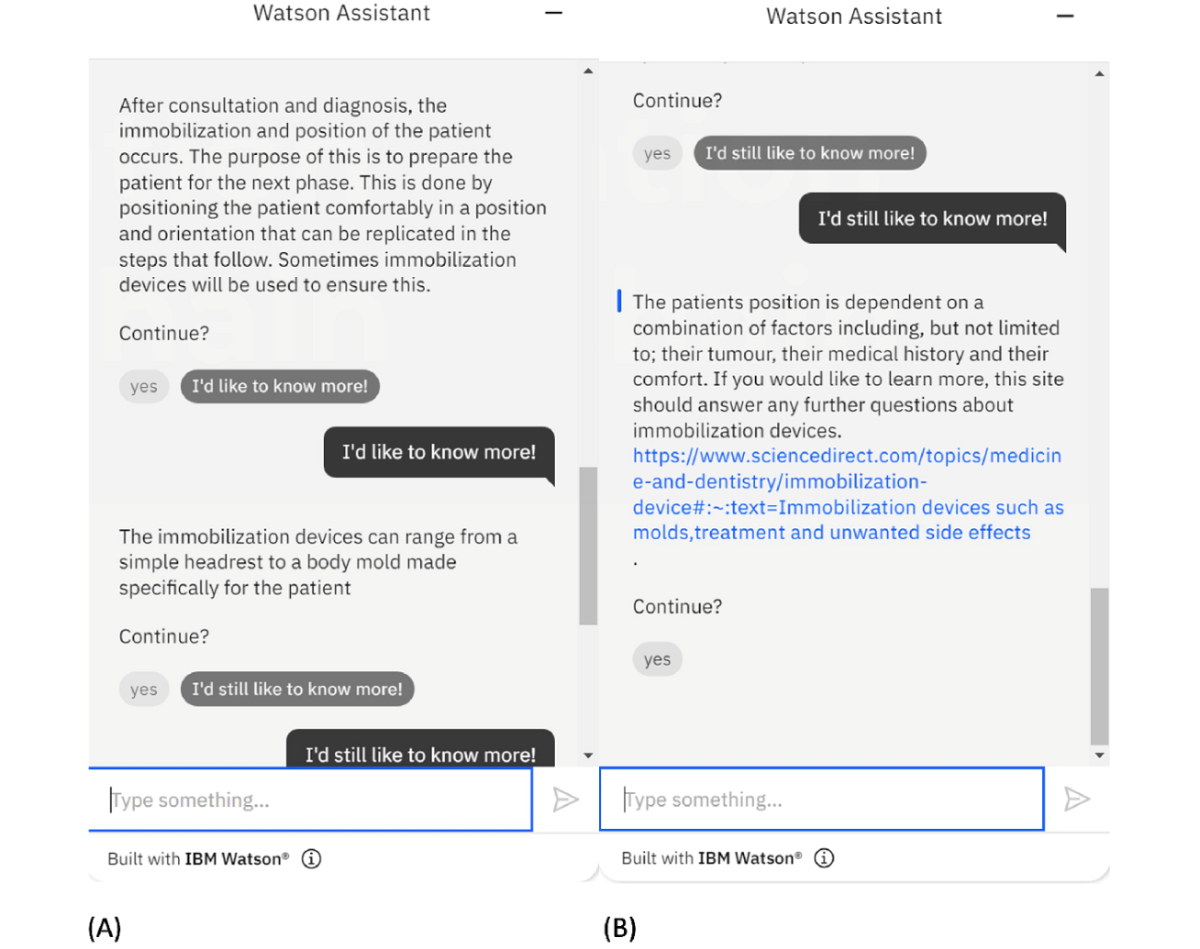
Responses of the bot when the user wants to have more information. (A) After the bot explains a procedure in the radiotherapy chain, the user is asked if they want to proceed to the next step or have more information. If the answer is “I’d like to know more!” the bot will provide more information. (B) After the information is provided, the bot will ask again if the user still wants to know more or continue; if the answer is “I still want to know more,” the bot will provide a link to the user so that they can assess more detailed information according to the topic from an external source.

When the bot has finished explaining a procedure in the radiotherapy chain such as consultation and diagnosis of a patient with cancer, as shown in Figure 5A, the user will have the option to select continuing to proceed to the next step in the treatment process or to have more information. If the user selects the latter, the bot will provide more information about the topic and ask again if the user still wants to know more. When the user answers that they would still want to know more, the bot will provide a link, so that the user can use it to access more related information on the internet, as shown in Figure 5B. A connection to other websites for more information allows the bot to maintain simplicity and update from the user’s response.

**Figure 6:**
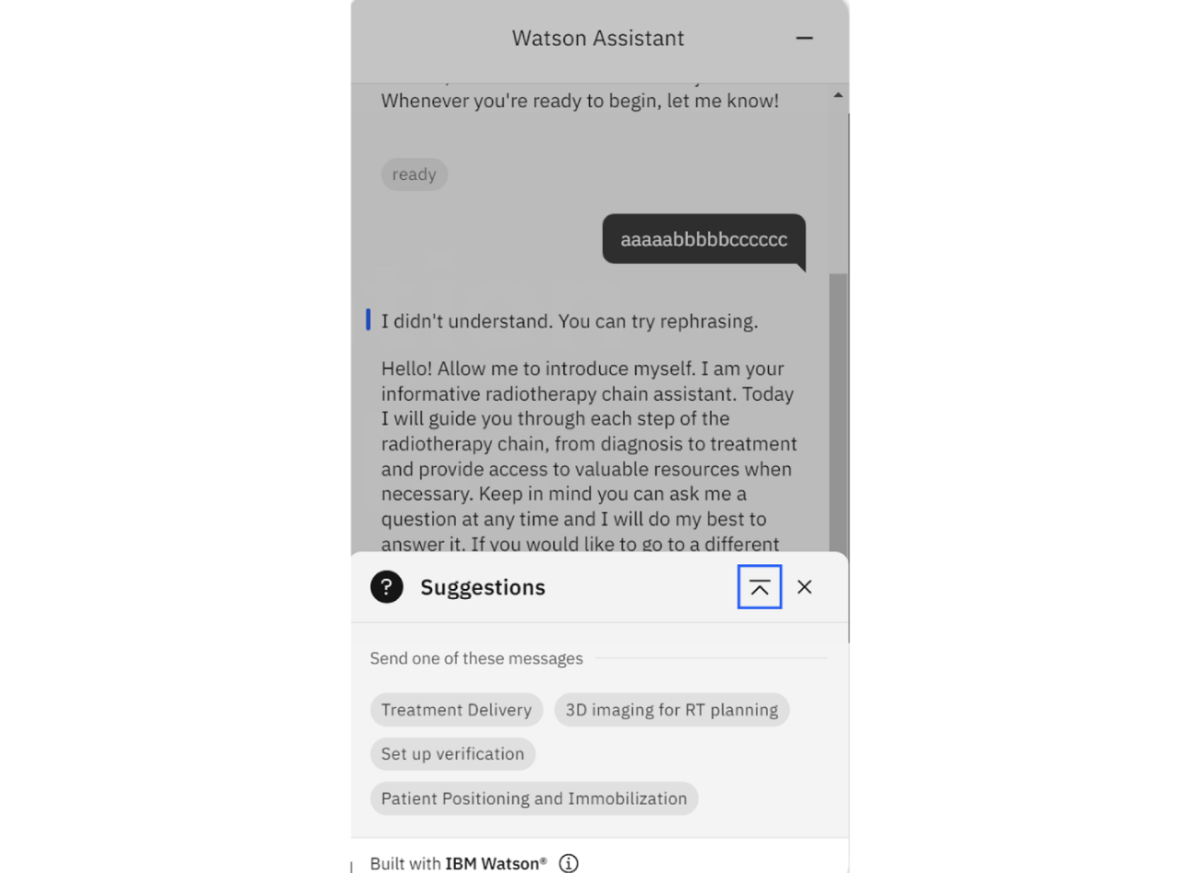
When the bot cannot receive a predictable response from the user, guidance will be provided with a list of items to select from. This can help the user to proceed to the next step even if they cannot answer well in the communication. Alternatively, the user can remove the window of suggestions and provide an answer to the bot again by rephrasing the original answer.

When the bot receives unpredictable response from the user, for example, a random string of letters (eg, “aaaaabbbbbcccccc”) instead of expected answers such as “ready,” “yes,” and so on, the bot will provide another window of suggestions to guide the user to answer the question (Figure 6). In that window of suggestions, the user only needs to select what they want to know from a list of items. Alternatively, the user can remove that window and answer the question again by rephrasing the answer. This can avoid incorrect communication between the bot and user when the latter cannot answer well (eg, typographical error in the answer). The guidance can also help the user to communicate with the bot when they cannot understand the question in the conversation.

### Performance Evaluation

The performance of the bot was evaluated randomly by a group of 50 people from the public. The participants were anonymous and unrelated to the development of the bot. The evaluation was based on three criteria including (1) information quality, (2) user experience, and (3) navigability. The score ranges from 1 to 5, corresponding to *poor*, *unsatisfactory*, *satisfactory*, *very satisfactory*, and *outstanding*, respectively. Here, *5* is the highest score, which indicates that the participant justified the bot as outstanding, whereas *1* is the lowest score, which indicates that the participant considered the bot as poor. The averages of the identified metrics were as follows: information quality=4.3 (SD 7.6%), user experience=3.6 (SD 5.2%), and navigability=4.7 (SD 4.1%). All metrics scored >3 (between satisfactory and very satisfactory), and both information quality and navigability scored >4 (between very satisfactory and outstanding). The scores indicated that participants in this evaluation were at least satisfied with the performance of the bot.

## Discussion

### Principal Findings

The aim of this study was to create a virtual assistant chatbot that can explain the radiotherapy chains to the user. This bot was designed using the help of IBM’s Watson Assistant. Once built, it will be incorporated into a resource that can be easily accessed by the general public. This chatbot was successfully created and hosted on an independent website [28]. The basic chain communicates about radiotherapy chains and their purpose to the user, with the ability to answer a few programmed questions.

The bot encountered very few issues during testing and was rated highly by users regarding navigability and quality of information. Inspecting the chat logs revealed that most users simply went through the root dialogue chain without requesting more information. The average score of 4.3 for information quality indicates that the bot’s primary purpose is adequately served, as this indicates an improved understanding of the radiotherapy chain. The lowest of the 3 scores was found to be 3.6 for user experience. This was potentially owing to the limited interactions that the user has with the bot and the limited variability in content. The score for navigability, despite being high, does not necessarily indicate the success of *jump to* commands or restart commands.

### Future Directions

As mentioned in the *Introduction* section, the IBM Watson’s API makes it easy for the programmer to modify the underlying structure or intents at any given time and add additional nodes to the conversation. As such, the successful functionality of the parent chain suggests that this bot can be improved with very few changes to the existing nodes. Adding additional root nodes could increase the number of relevant questions. With the addition of a multitude of nodes and the use of nodes with multiple conditioned responses, the bot could also be used for patient-specific information. For example, if a patient was diagnosed with cancer and had already progressed through their initial consultation, the bot could give the patient a more specific description of their remaining treatment based on the patient’s circumstances. Similarly, the first node could ask the user the reason for their interest in the radiation treatment process and use a tailored dialogue chain based on their response. This would provide a more user-specific experience. Alternatively, combining this bot with other chatbots or creating other dialogue chains that discuss different topics in radiotherapy could generalize the user experience.

### Limitations

There are a variety of unavoidable limitations in a bot of this nature. Currently, it will not be able to answer any questions outside the scope of radiotherapy and will not recognize unpredictable responses because this is the next stage of the configuration. An example is shown in Figure 6. By providing a list of items to select in case of communication impasse, the user can choose one of them to return to the scope of the bot and be back on track of communication. Another way to improve the limitation of the scope of the bot is to link it to an external website for further information. This can maintain the database of the bot as simple and comprehensive but can still satisfy the user if they want to have more information beyond the ability of the bot. Regarding the miscommunication of the bot when users entering unpredictable responses, the user can send feedback about the issue to the programmer of the bot. Then, the programmer can modify the bot to solve the problem in the routine update. It should be noted that comments and feedback from users are significant sources to improve the bot and reduce its limitations continuously.

### Conclusions

An education chatbot was created for patients, their family members, and the general public to understand the basic radiation treatment process by navigating the radiotherapy chain. Self-reported scores for information quality indicate that it also successfully conveys the information to the user in a manner that can be understood by the general population. In addition, the ease with which this bot can be improved and the potential uses of a chatbot of this nature establish it successfully as an effective proof of concept for informative chatbots in health care. The chatbot can successfully navigate its users through a radiotherapy chain. Current studies on public opinion and continuing improvements in NLP, ML, and internet-based technologies [29] indicate that these chatbots will become more advanced. Health care chatbots such as the one described in this paper have great potential [30], as health care chatbots can surpass humans in both speed and accuracy. Future studies include continuously updating the bot based on the users’ feedback. Thus, the performance of the bot can be improved from its preset process.

## Abbreviations

API: application programming interface
CT: computed tomography
ML: machine learning
NLP: natural language processing
